# Elite Ice Hockey Players’ Well-Being: A Scoping Review

**DOI:** 10.3390/sports13070225

**Published:** 2025-07-09

**Authors:** Pierre-Luc Veillette, Stéphanie Girard, Jason D’Amours, Vincent Huard Pelletier, Paule Miquelon

**Affiliations:** 1 Département des Sciences de L’activité Physique, Université du Québec à Trois-Rivières, 3351 Bd des Forges, Trois-Rivières, QC G8Z 4M3, Canada; stephanie.girard3@uqtr.ca; 2 Département de Psychologie, Université du Québec à Trois-Rivières, 3351 Bd des Forges, Trois-Rivières, QC G8Z 4M3, Canada; jason.damours@uqtr.ca (J.D.); paule.miquelon@uqtr.ca (P.M.); 3 Département des Sciences de L’éducation, Université du Québec à Chicoutimi, 555 Bd de l’Université, Chicoutimi, QC G7H 2B1, Canada; vhpellet@uqac.ca

**Keywords:** psychological factors, mental health, well-being, ice hockey players, coping, motivation, anxiety, depression

## Abstract

As mental health has gained prominence in recent years, elite ice hockey players have shared their experience of psychological problems, including adverse alcohol use, anxiety, depression, distress, eating disorders, and sleep disturbances. Mental health remains a sensitive issue for ice hockey players, as stigma, a strong hockey culture, lack of mental health literacy, and negative past experiences with seeking help constitute barriers to seeking support. This scoping review aims to identify the psychological factors contributing to elite ice hockey players’ well-being. After screening the titles and abstracts of three databases within a 2002–2025 timeframe, a total of 517 articles were retrieved. Seventeen articles targeting ice hockey athletes over 14 years of age competing at an elite level were selected. Three main categories emerged from the included studies: anxiety and depressive symptoms, motivational variables, and coping strategies at different career stages. Factors such as retirement, concussions, social support, parenting style, task-approach goals, and task-oriented behavior were influential components to the well-being and mental health of elite ice hockey players. Using the Lazarus and Folkman model, the identified psychological factors may help athletes and various actors surrounding them to better understand athletes’ well-being.

## 1. Introduction

Elite athletes competing in high-level sports face multiple challenges that affect their mental health [1]. Moreover, they are less likely to reach out for help [2] and report a lower level of well-being than the general population [3]. Male athletes are more likely to display impulsive behaviors (e.g., use of psychoactive substances, alcohol), while female athletes are more vulnerable to developing eating disorders [4]. Specifically, 16 to 34% of current and former elite athletes exhibit mental health symptoms, including sleep disturbances, depression, anxiety, and general psychological distress [5]. Additionally, it is suggested that, for these athletes, the onset of mental health problems mainly occurs between 17 and 21 years of age [6]. Indeed, it is a time when they face intense mental and physical demands because of the overlap between the peak age when athletes as a whole are at risk of developing mental disorders and the peak competitive years of elite athletes [1]. Given the unique demands of elite sports on young athletes who must also navigate the same developmental obstacles as other adolescents and young adults, more studies are needed to study this population. This scoping review focuses on elite ice hockey athletes because of the growing concerns regarding the mental health of this population in North America and Europe [7,8]. This review aims to meet the need of gathering information for this specific group of elite athletes in which they must deal with high pressure and demands daily, therefore impacting their mental health and well-being [2].

Indeed, more than 513,000 Canadians and 550,000 Americans practice ice hockey [9]. In the 2023–2024 National Hockey League (NHL) season, Canadian players made up over 40% of the league, while American players accounted for almost 30% [10]. The mental health of elite athletes has gained significant academic attention in recent years [7,8]. Furthermore, elite hockey players have drawn media focus concerning various mental health issues [11,12,13,14]. Many current and retired professional hockey players have shared their experience of psychological problems, including adverse alcohol use, anxiety, depression, distress, eating disorders, and sleep disturbances [15,16]. Mental health nevertheless remains a sensitive issue for ice hockey players, as stigma, a strong ice hockey culture, the lack of mental health literacy, and negative past experiences of help-seeking constitute barriers to help-seeking behaviors [2,17]. The hypermasculine culture in ice hockey fosters stereotypes about masculinity and toughness, discouraging emotional expression, which perpetuates a culture of silence regarding mental health issues [18,19,20]. Although the challenges related to such psychological problems across different ages are unclear, known risk factors include the pressure to perform, the high expectations of others, and homesickness [21].

### 1.1. Elite Athletes’ Well-Being

Literature contains many theoretical definitions of well-being. The concept of well-being is commonly understood as comprising two main components dating back to Aristotle’s time: hedonic and eudaimonic well-being [22]. Hedonic well-being refers to the pursuit of pleasure and the avoidance of pain, emphasizing the presence of positive emotions and overall life satisfaction. In this regard, Diener [23] significantly contributed to the field with his work on subjective well-being and later developed the five-item Satisfaction with Life Scale (SWLS) to assess individuals’ cognitive evaluations of their lives [23]. In contrast, eudaimonic well-being centers on self-realization and the pursuit of a meaningful life [24]. According to Carol Ryff [25], psychological well-being consists of six key dimensions: autonomy, environmental mastery, personal growth, positive relations, purpose in life, and self-acceptance [25]. Furthermore, Ryan and Deci [26,27], through their Self-Determination Theory, identified three fundamental psychological needs explaining eudaimonic well-being: autonomy, competence, and relatedness.

From the athletes’ point of view, the definition of well-being targets eight specific components: confidence and self-worth, personal balance, aligned purpose, fulfillment, performance, personal agency, psychologically safe relationships, and psychological adaptability [28]. These components collectively extend the current knowledge on eudaimonic well-being, as it focuses on a holistic view of the athletes’ reality.

The Dictionary of Psychology of the American Psychological Association (APA) defines it as a “state of happiness and contentment, with low levels of distress, overall good physical and mental health and outlook, or good quality of life” [29]. In other words, well-being emphasizes positive psychological states and an absence of negative cognitions and feelings [30]. Consistent with the systematic review by Agnew et al. [31], this paper considers well-being as a state of mind and as an indicator of good mental health. Specifically, the authors studied athletes’ well-being with regards to two themes: sports environment and adversity, both of which have a positive and negative component. For example, participating in sports culture can provide meaningful experiences, where a high level of support and personal growth contribute to higher levels of well-being. However, sports environment and adversity can contribute to lower levels of well-being thanks to factors such as homesickness, external and internal pressure, lack of confidence, and injuries. Because there has been no research to date on factors contributing to the well-being of elite ice hockey players, Agnew’s review highlights the importance of examining this subject.

### 1.2. Theoretical Framework

This review explores the psychological factors affecting the well-being of elite ice hockey players through the lens of the transactional model of stress and coping by Lazarus and Folkman [32]. This model captures the fluid nature of stress in sports, influenced by performance pressure, injuries, career transitions, and social support. Its core elements—primary appraisal (perceived stressor), secondary appraisal (perceived resources), and coping responses—provide insight into how psychological factors like anxiety, motivation, and self-regulation are interconnected processes impacting well-being. This framework helps to identify and organize relevant psychological factors across different stages of athletic development.

The first category involves primary appraisal, during which the athlete evaluates the significance of external stressors. These may include performance pressure, interpersonal conflicts with coaches or teammates, and the physical and psychological demands inherent to elite sports. The athlete determines whether these stressors are irrelevant, positive, or threatening. When perceived as threats to performance or well-being, such stressors can have harmful consequences on mental health. The second category refers to secondary appraisal, which concerns the individual’s perceived ability to manage the situation. Personal characteristics—such as resilience, perfectionism, intrinsic or extrinsic motivation, and perceived competence—shape this evaluation. These internal resources influence how the athlete interprets the stressor. For instance, a player with high emotional stability and intrinsic motivation may view a high-stakes competition as a stimulating challenge. At the same time, a less confident athlete may interpret the same situation as a threat. The third category includes coping strategies employed to regulate stress. Problem-focused strategies, such as skill development or seeking support, aim to directly address the source of stress. In contrast, emotion-focused strategies, such as mindfulness and emotional regulation, help manage emotional responses to stressors. The transactional model provides a comprehensive lens for analyzing stress among elite ice hockey players by clearly distinguishing between primary appraisal, secondary appraisal, and coping strategies.

### 1.3. Factors Linked to Well-Being

Ice hockey players must deal with sports injuries, which would mainly correspond to primary appraisal of the transactional model [32]. Many researchers have examined the relationship between sports injuries and psychological factors. Indeed, athletes’ psychological problems tend to increase along with the number of injuries and surgeries [33]. Because of the previously mentioned hockey culture associated with stigma, hockey players may feel socially pressured to play, despite being injured [34]. A history of concussions, moreover, raises the risk of developing symptoms of distress that worsen with each new episode [35]. In addition, hockey players who suffered from concussions during their career described a social isolation that led to depressive symptoms [36]. Once they transition to their post-career, they may feel unprepared to deal with their injuries and symptoms of distress [37,38]. Furthermore, career-ending injuries add to the struggles of transition to retirement, along with financial, relational, and social problems regarding rehabilitation [39]. To improve rehabilitation following an injury, research suggests encouraging athletes to discuss the issue with their injured teammates and listen to how they cope with relational ruptures with their teammates, coaches, managers, and families [37]. Social support, athletes’ attitudes toward recovery, and coaches’ role during rehabilitation are some factors that promote recovery [40]. These avenues are especially relevant, since sports injuries are associated with lower levels of well-being in athletes [41].

In addition to sports injuries, research highlights other factors that affect elite athletes’ well-being, such as athletic identity [16,42,43,44] and the relationships or roles of parents and coaches [45,46,47,48]. Furthermore, the perceived value of elite sports has been shown to positively impact individual subjective well-being [49]. However, the high demands and intense training loads associated with competitive sports create significant pressures for elite athletes, posing potential risks to their mental health [50]. The elite sports environment exposes athletes to unique stressors, including performance expectations, organizational challenges (e.g., frequent travel, homesickness), and personal issues (e.g., family conflicts) [50,51]. Over time, these stressors can lead to sports burnout, where the cumulative demands of elite sports take a toll on athletes’ well-being. Ultimately, these challenges significantly increase the risk of mental health issues among elite athletes [50,52].

With the issues outlined above, a recent scoping review on elite youth athletes provided great insight on different realities these young athletes are up against [53]. By screening a population with a mean age ranging from 12–17 years old, their findings illustrated that anxiety and depression, burnout symptoms, substance misuse, and aspects of well-being are factors associated with mental health in elite athletes participating in various sports. However, none of these studies focused specifically on elite ice hockey players. Moreover, ice hockey represents a particularly demanding sport due to its unique combination of physical, mental, and environmental challenges associated with its competitive context [2,17]. The present review aims to fill the gap on athletes’ well-being in this specific sport [54] and to explore the situation regarding this group of athletes in line with the transactional model of stress and coping developed by Lazarus and Folkman [32].

## 2. Materials and Methods

This scoping review was developed based on the PRISMA-ScR guidelines (Supplementary File S1) and is consistent with the review protocol and the Arksey and O’Malley [55] five-step process, which involves: (1) identifying the research question; (2) identifying relevant studies; (3) study selection; (4) charting the data; and (5) collating, summarizing, and reporting the results.

### 2.1. Identifying the Research Question

As the objective of this scoping review was to identify the psychological factors contributing to ice hockey players’ well-being, there was only one research question: What psychological factors contribute to the well-being of elite ice hockey players?

### 2.2. Identifying Relevant Studies

**Figure 1:**
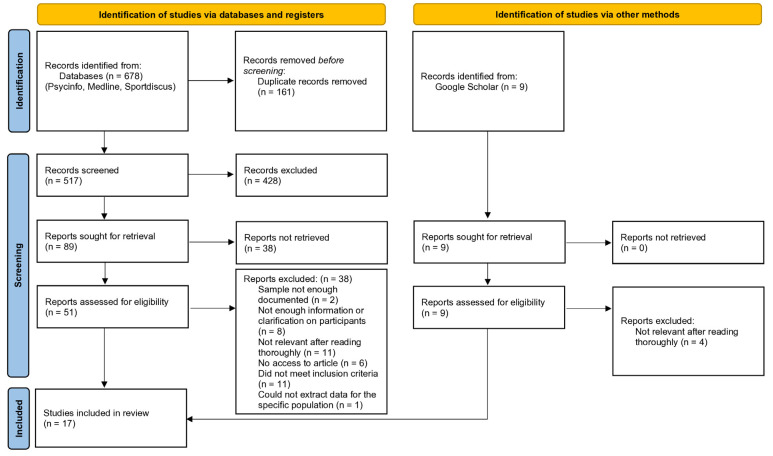
PRISMA flowchart.

The initial search for potential articles was conducted 11–30 May 2022, generating a total of 687 articles. The original search timeframe targeted the past 20 years (2002–2022). However, since the writing process required more time than initially planned, the search was updated three times to include newly published articles, which extended the timeframe to 2025. Studies before 2002 were not considered to ensure that the review focused on practices, conceptual frameworks, and societal contexts more closely aligned with modern settings. As shown in Figure 1, the following databases were systematically searched: Psycinfo (138), Sportdiscus (218), and Medline (322). Potential sources considered relevant in GoogleScholar (9) were retrieved manually and also analyzed, bringing the total number of articles to 687. With the assistance of the university’s specialized librarian, key words used for the literature search included the following: psychological factor* OR anxiet* OR motivation* OR *confidence OR “self concept” OR “self esteem” OR passion* OR pressure OR resilience OR “emotional intelligence” OR coping OR “psychological adjustment*” OR “well being” OR “well-being” OR wellbeing. Descriptors were also added to target specific concepts in the literature search: DE “performance anxiety” OR DE “Self-esteem” OR DE “self-confidence” OR DE “psychological resilience” OR DE “psychological stress”. Moreover, we used the key words “ice hockey” OR “hockey player*” to obtain and limit articles to ice hockey only. Because no advanced settings were available for Google Scholar, a manual search was conducted using the key words “psychological factors” and “ice hockey” within the 2002–2025 timeframe and was completed by consulting reference lists.

In all three databases (PsycInfo, Medline, and Sportdiscus), the search terms were applied to titles and abstracts. These field specifications were kept consistent across databases to ensure the retrieval of comparable articles from each source. For GoogleScholar, due to its more limited search functionalities, we applied the full search equation and restricted the results to articles published between 2002 and 2025.

Regarding the article selection process, two authors (PLV and JD) independently reviewed all articles identified through the literature search, assessing their relevance based on predefined inclusion and exclusion criteria. Following the PRSIMA guidelines, each author initially selected potential articles for inclusion in the scoping review. Following this, they compared their selections and discussed the relevance of each article that passed the initial screening. In cases of disagreement, the authors revisited the specific articles and engaged in discussion to reach a consensus. Although a third author (SG) was available to arbitrate unresolved conflicts, no such intervention was necessary, as all disagreements were resolved through discussion between the two primary reviewers.

#### 2.2.1. Inclusion Criteria

The inclusion criteria considered for this review included peer-reviewed articles published between 2002 and 2025. In addition, interest in the mental health of elite athletes and research involving hockey players increased in the last 20 years. In these studies, potential participants had to be considered “elite”, which meant they were required to compete in professional leagues or in the most competitive leagues for adolescents. Thus, participants had to be at least 14 years old. For example, the inclusion criterion was met by the M18 AAA, which features the best players (mostly between the age of 15 and 18 years old) in all of Quebec’s (Canada) hockey leagues. However, the inclusion criterion was not met for a high school team, since the league level was not considered high enough. Articles on psychological factors and ice hockey players were selected. A few examples of psychological factors were the nature of significant childhood and adult relationships, the experience of ease or stress in social environments (e.g., school, work), and the experience of trauma [56]. Finally, articles examining different sports using the same samples were not included unless conclusions could be drawn based solely on the sample of elite ice hockey players.

#### 2.2.2. Exclusion Criteria

Studies that included participants under the age of 14 were excluded. As for the competition level, this criterion was defined by the best available leagues for a given territory. Studies failing to consider this for their sample did not meet our inclusion criteria. Articles focused on the point of view of coaches, parents, or fans were also excluded, as were dissertations and theses, given they are not peer-reviewed. Additionally, studies were excluded if they were: (a) written in a language other than English or French, (b) published before 2002, and/or (c) not empirical.

### 2.3. Study Selection

Abstracts and titles were screened first. After duplicates were removed from the 687 articles first selected from the databases, a total of 517 potential articles were screened by retaining only those that met the inclusion criteria. The nine articles retrieved manually from Google Scholar were assessed for eligibility, and five articles were considered relevant for this current scoping review. During screening, articles were divided into three categories: (1) those relevant to the research subject; (2) those the researchers were unsure of; and (3) those considered irrelevant. A second reviewer systematically examined and screened the articles using the same process. Both reviewers then discussed any disagreement and, if unable to reach a consensus, asked a third reviewer to make the final decision.

### 2.4. Charting the Data

Homemade analysis grids were used to provide a systematic method for reading articles in depth. The main characteristics of the 51 articles (study objective, population, scales and variables, statistical analysis, conclusion, and limitations) were observed to validate their relevance. A column with the heading “Decision” was added to make the final decision. Overall, 12 articles were selected from the databases, as well as 5 studies from Google Scholar, which led to the selection of 17 articles for the current scoping review that met every inclusion criterion. Various themes emerged from the studies, becoming subjects of further analysis to determine similarities and differences, as presented in Table 1.

### 2.5. Collating, Summarizing, and Reporting the Results

The theoretical model also guided the interpretation and categorization of findings throughout the review process. Stressors such as concussions, transitioning out of the sport, or team selection experiences were mapped onto the primary appraisal process, as they represent events perceived as significant by athletes. Variables such as motivational climate, parenting style, and perceived competence influenced the secondary appraisal, which reflects the athlete’s judgment of their coping capacity. Finally, strategies such as seeking social support, emotional regulation, or engaging in performance routines were associated with coping responses. By structuring the results in this manner, the model enabled a more nuanced understanding of how psychological well-being in elite hockey players is shaped by appraisal processes and coping resources over time.

Thus, the selected articles were presented in a table to indicate their similarities and differences. Themes were identified to meet the objective of the scoping review regarding the implications for research and the theoretical framework. Consistent with the guidelines [54,74], there was no quality evaluation of the selected studies. Results were interpreted by the authors and are described later in the article.

## 3. Results

### 3.1. Descriptive Statistics

Of the 687 papers yielded from the search strategy, 517 abstracts and titles (74%) met the inclusion criteria. After the full-text reading stage, the list was reduced to 17 peer-reviewed articles retained for further analysis (see Table 1). Years of publication ranged from 2005 to 2024, with seven (41%) articles published between 2005 and 2010.

One study only focused on women [61]. In terms of different age groups, nine (53%) studies discussed ice hockey players aged 13–21 years [57,60,62,64,65,66,70,72,73], while four (24%) focused on adult ice hockey players over 21 years old [58,59,68,69], and three (18%) examined both adult and adolescent ice hockey players [61,63,67,71].

The picture between North American and European studies was balanced in terms of study location. There were eight (47%) North American studies [57,58,59,60,62,68,70,73], eight (47%) European studies [61,63,64,65,66,67,71,72], and one (6%) study including ice hockey players from both continents [69].

As for the sample size of the studies in this review, six (35%) consisted of 1 to 25 participants [59,60,61,68,69,71], five (29%) of 26 to 250 participants [57,63,64,70,72], four (24%) of 250 to 500 participants [58,62,65,67], and two (12%) of more than 501 participants [66,73].

Finally, 11 (65%) of the 17 studies used quantitative surveys [57,58,62,63,64,65,66,67,70,72,73], while the 6 others (35%) relied on a qualitative methodology (i.e., interviews) [59,60,61,68,69,71]. Most of the included studies employed descriptive statistics to summarize their data, alongside inferential methods such as *t*-tests, ANOVAs, and ANCOVAs. Additionally, correlational and regression analyses were frequently considered to examine relationships between variables and to support more in-depth interpretation of the data [57,63,65,66]. Finally, out of the seventeen included studies, ten were cross-sectional designs [57,58,63,64,65,66,67,70,72,73], six were different types of interviews (retrospective, semi-structured, phenomenological) [59,60,61,68,69,71], and one was a longitudinal study [62].

### 3.2. Secondary Appraisal Variables

The 17 studies in this scoping review covered a wide range of psychological factors that can contribute to ice hockey players’ well-being, including anxiety and depressive symptoms, motivational variables, and coping strategies observed at different career stages.

### 3.3. Anxiety and Depressive Symptoms

As shown in Table 1, three studies quantitatively measured depressive and anxiety symptoms using validated scales [58,63,64]. One study revealed that retired ice hockey players experienced more depressive symptoms than active ones [58], whereas Géczi et al. [63] showed that older adults experienced a more positive affective state from the standpoint of anxiety and stressful situations, compared to younger participants. Finally, no difference was observed between the affective state and symptoms of anxiety/depression among U16 (players under the age of 16), U18 (under 18), and U20 (under 20) ice hockey players [64].

### 3.4. Motivational Variables

Nine studies examined motivational variables (e.g., passion, motivational climate, self-determination, need satisfaction), and all did so quantitatively, using validated instruments [57,61,62,65,66,67,70,72,73]. Task-approach goals (competence and satisfaction derived when a person learns new skills, improves their performance, and does their best [75]) were linked to a higher level of enjoyment experiences [65], perfectionistic strivings [72], and passion [73]. Similarly, positive parenting (i.e., authoritative parenting, which is characterized by warmth, clear boundaries, and open communication [76]) was associated with task-oriented behavior and higher satisfaction in ice hockey [66]. Moreover, a task-involving climate (i.e., individuals evaluated on the basis of their personal development, rewarded for effort and individual improvement, and placed in mixed-ability groups [75]) was strongly correlated with enjoyment [65].

At the individual level, Spink et al. [70] suggested that when participants perceive their roles to be clear and feel they can express themselves, they will put more effort into various team performance situations. Moreover, being selected or not for a highly competitive team also influenced positive and negative affective states. For instance, athletes selected for the highly competitive team experienced a substantial decrease in negative affectivity, whereas those not selected maintained their high levels of negative affect after the selection process [62]. Also, passion was found to be a supportive and motivating factor linked to happiness [61] and better psychological adjustment [57] when hockey players’ passion type (i.e., obsessive passion) aligned with their environmental demands (i.e., higher competitive league).

From a collective perspective, Nassi & Nagy [67] identified four distinct motivational profiles based on their levels of self-determined motivation. The results showed that players with higher self-determined motivation reported better coping skills and lower levels of competitive anxiety and perceived a more positive, task-oriented motivation climate. On the opposite side, players with lower self-determined motivation experienced weaker coping abilities, higher anxiety, and perceived a more ego-oriented, less favorable climate.

### 3.5. Coping Strategies

On one hand, five qualitative studies assessed coping strategies observed at different career stages [59,60,68,69,71]. Regardless of age group or playing level, participants in all five studies reported that a good social support network, including family, friends, and coaches, was a useful external coping resource and led to positive developmental outcomes. Furthermore, ice hockey players who reached the NHL shared the different psychological skills and characteristics they acquired and developed from a young age. As they progressed through higher levels of the game, players developed more performance-oriented psychological skills (e.g., emotional regulation) and psychological characteristics (e.g., reframing adversity as a challenge), which are pivotal components in career development [69]. They then expanded and refined coping strategies to maintain acquired psychological skills, such as visualization and self-talk [69]. Battochio and Stambulova [59] highlighted that for NHL rookies, readjusting their expectations and working hard led to greater confidence and personal enhancement.

Conversely, reduced playing time was found to decrease young male athletes’ levels of self-confidence, which negatively impacted their personal growth off the ice [60]. Transitioning within different career stages (e.g., junior to senior) was also found to be mentally demanding, as it is difficult to remain motivated and determined when facing contextual barriers, such as distorted expectations, lack of confidence, lack of opportunities, or injuries [71]. As for transitioning out of their hockey career, a lack of coping resources and the loss of the sense of identity reinforced by fans may add to players’ struggles during retirement [68].

## 4. Discussion

This scoping review examined psychological factors affecting the well-being of elite ice hockey players using Lazarus and Folkman’s transactional model [32]. Following Arksey and O’Malley’s five-step process [55], 17 relevant articles were identified based on specific criteria, categorized into anxiety and depressive symptoms, motivational variables, and coping strategies at various career stages. Anxiety symptoms are common among elite players, as they strive for peak performance while managing mental health. However, as Géczi et al. [63] noted, these athletes develop better coping mechanisms with experience, improving their ability to handle high-pressure situations as they age.

Retired ice hockey players face a higher risk of developing depressive symptoms compared to active players [16,58], as noted in recent literature on retirement challenges [68]. This increased risk may stem from a loss of perceived athletic identity, leading to confusion about their roles as “regular citizens”. A study by Gouttebarge et al. [5] indicated high levels of anxiety and depression among elite athletes, with retired players experiencing more severe symptoms than the general population. According to Lazarus and Folkman’s model [32], these symptoms may arise from how players evaluate their coping resources post-retirement, potentially intensifying their distress. This cycle can diminish psychological functioning. Social support and a positive motivational climate can help players view stress as a challenge rather than a threat.

These mental health issues are explained by a wide range of factors, including a history of concussion among participants. Caron et al. [77] highlighted the impact of diagnosed concussions among retired NHL players who also experienced anxiety and depression. The authors discovered that these athletes remain significantly affected by such symptoms post-career, often experiencing isolation, depression, and even thoughts of suicide [78]. As suggested by Stephan and colleagues [79], it may take up to a year for the well-being of retired athletes to improve in the face of challenges that include feelings of loss or emptiness. More specifically, the absence of social support may play a key role during the period of transition to retirement following an injury, as previously established by the work of Alfermann et al. [39].

A key finding on motivational variables is that adopting task-oriented goals and behaviors leads to positive outcomes like enjoyment and passion. This aligns with previous research, as task-oriented goals focus on competence and satisfaction through skill development and effort [80]. Elite athletes, such as ice hockey players, are motivated by personal improvement, enhancing their enjoyment and well-being. This drive comes from their experiences in pursuit of maximum potential in their sport [81]. Moreover, a higher level of self-determined motivation in competitive athletes underlines the importance of psychological well-being and performance in sports [67]. Similar patterns are observed in other elite sports like skiing, where task-oriented behaviors foster a belief in success [82].

When considering the significance of a motivational climate for athletes’ well-being and satisfaction in elite sports, the role of parenting style emerges as crucial. Authoritative parenting—characterized by warmth, clear boundaries, and open communication [76]—plays an essential role in fostering task-oriented behavior and enhancing satisfaction among ice hockey players. As children mature and gain more experience in the sport, they increasingly desire autonomy and independence in making sports-related decisions. The introduction of responsibilities at this stage serves to boost their motivation. This approach to parenting not only nurtures self-discipline and responsibility in young athletes but also helps to preserve a positive parent–child relationship within the realm of ice hockey [68]. By consistently practicing an authoritative parenting style and adjusting their involvement as necessary, parents can significantly contribute to their child’s long-term satisfaction and success in the sport while simultaneously reinforcing the child’s motivation to pursue their passion [66]. This finding demonstrates that parents and the social support they provide are of vital importance for elite ice hockey players [58,60]. Finally, the motivational aspects would also be associated with the secondary appraisal of Lazarus and Folkman’s [32] model. Indeed, task-approach goals, task-oriented behavior, and the motivational climate are linked to hockey players’ well-being, as they will be more likely to perceive themselves as capable of handling competitive demands and psychological stressors in a more supportive, motivational climate.

Continuing along these lines, the impact of coaches and the climate they create on the lives of elite athletes must be taken into consideration, even though the issue was not covered in this scoping review. Interestingly, the role of coaches in the process of sport career termination was not considered significant in the study by Lagimodiere and Strachan [68], which contradicts other findings on the topic [59,60,69,71]. In a recent study [60], young players reported feeling overly criticized by their coaches, which led to a decrease in their confidence levels. In this case, the reason may be that coaches did not always engage with their former players effectively or did not use appropriate communication methods. It is therefore crucial for hockey players to develop effective coping mechanisms from a young age. For example, Andrijiw [44] suggested that the ice hockey organization plays a role in shaping individuals’ identities through their experiences with the team. Maintaining a relationship between teams and their former players could potentially alleviate identity struggles, especially for professional ice hockey players who must cope with injuries upon retiring.

The findings indicate that coping strategies play a crucial role at all stages of an athlete’s career, serving as adaptive mechanisms that not only help manage stress but also enhance the ability to navigate high-pressure situations and psychological challenges as players advance in their careers. According to Géczi et al. [63], this development is often essential for maintaining elite status, as the rigorous demands of ice hockey necessitate that players adapt and build resilience over time. Additionally, these coping strategies are vital for managing stress associated with performance, injuries, and retirement. For example, athletes who seek social support during injuries tend to cope better during their rehabilitation process [83]. Thus, strong support networks—including teammates, coaches, and family—are instrumental in fostering effective coping strategies. Moreover, alongside players’ adaptive mechanisms for managing stress and high-pressure situations, emotional regulation and performance-oriented psychological skills, as emphasized by Pankow et al. [69], closely align with the third appraisal of Lazarus and Folkman’s [32] model. By effectively regulating their emotions and developing psychological skills that enhance performance, hockey players are better equipped to utilize their coping strategies to meet the demands of an elite-level career.

Finally, studies by Gaudreau et al. [62] and Amiot et al. [57] highlighted some similarities when it comes to team selection. Indeed, they shed light on the importance of aligning with one’s passion type when being chosen for a team, as this will impact players’ affective state, positively or negatively. Thus, it is clear that the more a player feels they belong to the team, the greater their sense of well-being and, consistent with Spink et al. [70], the more effort they will exert in various team situations. However, it also highlights the emotional toll of the selection process and underscores the importance of supporting athletes’ mental well-being within the context of elite sports. In this regard, research conducted on readjusting expectations to succeed in sports supports the work of Battochio and Stambulova [59].

Many of the studies used well-established psychometric instruments to assess variables such as perfectionism, anxiety, and motivation [58,72]. As this would be considered a methodological strength, the variability in age within the selected studies, ranging from elite youths [63], to professional adults [58], and to hockey players in career transition [68], makes it very difficult to generalize the findings. However, these findings offer insight into the challenges that athletes may encounter at each stage of their career. Several studies underlined the importance of social support, which constitutes a common theme across selected studies [58,60,65,66]. Indeed, it was reported that social support is positively linked to motivation and psychological well-being. Continuing along these lines, psychological well-being is associated with goal orientation and coping [63,65,72], as mastery- and task-oriented behaviors led to positive emotional outcomes, and effective coping strategies helped deal against competitive anxiety. Finally, the selected studies used different design approaches, such as quantitative [66], qualitative [68], and experimental [58].

However, defining one’s expectations and standards starts early in life. Building and acquiring solid foundational assets lead to the later development of psychological skills that can benefit the team and allow the player to solidify their position. Indeed, the ability to deal with task-oriented components will help increase a player’s confidence, clearly an asset for those playing in the world’s most competitive hockey league. As noted by Pankow et al. [69], it would be interesting to identify and further explore the psychological skills and characteristics young athletes should acquire.

### 4.1. Avenues for Future Research

Only a single study among those we selected discussed the perspective of women, despite the growing number of women’s professional leagues [61]. Indeed, although many professional leagues worldwide support their athletes financially, only a limited number of professional women’s hockey leagues enjoy this privilege. Still, it would be worthwhile in the future to examine well-being within a sample that includes a more balanced representation of both women and men, especially with the Professional Women’s Hockey League (PWHL), which offers significant visibility and opportunities for elite women hockey players worldwide.

Furthermore, because anxiety and depressive symptoms, injuries, and post-career behaviors are known to impact well-being, future research would do well to focus on the risk and protective factors associated with well-being among elite ice hockey players. Studying younger samples and targeting risk factors could help create intervention programs to prevent symptoms before they develop.

Other important factors influencing well-being that should be considered are various forms of abuse/bullying, such as verbal harassment, physical abuse, and sexual misconduct. These ongoing and concerning issues within elite sports environments carry significant implications for athletes’ well-being. Future research should further investigate the long-term psychological, emotional, and social consequences of such negative experiences, as they relate to athletes’ mental health, identity development, and career trajectories. Sport organizations have an essential responsibility towards this matter and should prioritize the implementation of preventive measures, including reporting mechanisms or education programs, to create a safer environment. These measures should promote the importance of respect, integrity, and psychological safety, where elite athletes are supported both in their personal development and performance. By doing so, athletes will be ensured to reach their full potential while protecting their well-being and reducing the normalization of harmful behavior in elite sport settings.

Finally, note that the coping strategies in the articles included here were analyzed based on a qualitative methodology, which demonstrated that qualitative research is helpful for understanding three components, as underscored by Maxwell [84]: the meaning of the events, the context within which the participants act, and the processes by which events and actions take place. This highlights the importance of each player’s personal trajectory in navigating different psychological factors at various career stages. Therefore, research focused on similar issues should employ a qualitative or, at the very least, a mixed-methods approach. It is also worth mentioning that since not many studies used quantitative data, this methodology could be profitable to explore how such strategies relate to indices of well-being over time or in different populations.

### 4.2. Implications for Practice

To promote well-being among ice hockey players and increase athletes’ chances to improve performance, it is essential to offer positive and constructive feedback [62]. Challenges that can affect well-being may include reduced playing time or lower on-ice point production, as well as off-ice transitions, such as moving away from home [60]. Additionally, interventions should focus on athletes who are considered vulnerable (e.g., those with low need satisfaction, low self-determination, and a weak academic identity). Interventions such as these could help to educate and provide training for coaches in elite ice hockey programs.

Additionally, more attention should be given to support for players. Veterans’ greater involvement with younger players could help with the transitions across career stages [59]. Our results show that coaches have an impact on players’ development; hence, offering players appropriate support may create a more positive understanding of their role within the team [59,69]. Coaches could also utilize the empirical model of Stambulova et al. [71], titled “Phases in the junior-to-senior transition of Swedish ice hockey players,” to increase awareness and understanding of the process, reduce dropout, facilitate the proactive efforts outlined in the model, and create a supportive environment. This model could also be of benefit to sport psychology practitioners [71].

Finally, preparation and planning could improve the likelihood of a positive experience for ice hockey players close to retirement [68]. As an additional avenue of support, the *Life After Hockey and Athlete Career Transition Program* [85] is a good example. This program is designed to help current and former professional hockey players as they transition out of their career. In line with findings on the prevalence of anxiety and depressive symptoms, Aston et al. [58] also identified some programs and psychotherapies aimed at helping athletes cope with anxiety, depression, and alexithymia. For example, cognitive behavioral therapy and mindfulness-based interventions were found to help reduce depressive symptoms [58]. For active hockey players, programs to reduce the stigma surrounding mental health in ice hockey have been examined and found to be effective, not only to reduce stigma, but also to provide knowledge on mental health and support [86].

### 4.3. Limitations

This scoping review had certain limitations. First, it included elite ice hockey players only, which limits the generalizability of the sample to other sports or recreative hockey players. Indeed, the collected results of each study considered only data pertaining to ice hockey players and excluded mixed results (e.g., data were excluded when ice hockey players were studied alongside soccer players). Second, only one of the 17 studies included focused on women. The findings, therefore, cannot be generalized to women, as only one sample from literature met our inclusion criteria. Third, the literature search could not encompass all possible keywords related to psychological factors within the equation. This limitation may have narrowed our research to studies that contained specific keywords only; thus, we used keywords that were most relevant and, therefore, most likely to appear in the existing literature. Such challenges with this literature search mean that it is possible that some studies were missed and, therefore, not included as potential articles in this current scoping review. Fourth, the language criterion may have excluded some articles from, for example, European countries where English was not the language used.

## 5. Conclusions

Our scoping review allowed for the identification of various psychological factors and coping strategies contributing to the well-being of elite ice hockey players. Specifically, anxiety and depressive symptoms, motivational variables, and coping strategies within different career stages were categorized as the main components regarding well-being in ice hockey players. This scoping review may help players, coaches, practitioners, and hockey organizations to increase awareness on well-being in providing insights on the matter.

## Figures and Tables

**Table 1:** List of included studies.

	Citation Information	Demographic Information	Location of Study	Methodology	Variables	Key Findings
1.	Amiot et al. [57]	*n* = 233 Age range: 13 to 18 All men	Québec, Canada	Quantitative (survey)	Motivational constructs	Highly competitive leagues lead to a higher psychological adjustment than less-competitive leagues. Ice hockey players that are obsessively passionate will have a higher psychological adjustment if selected in highly competitive leagues, compared to harmonious players. Conversely, psychological adjustment will be higher for harmonious players if selected in less competitive leagues, compared to obsessive, passionate players.
2.	Aston et al. [58]	Active players *n* = 175 *M_age_* = 27.3 *SD* = 4.15 Retired players n = 234 *M_age_*= 35.6 *SD* = 6.94 All men	Canada and United States	Quantitative (survey)	Anxiety and depression symptoms	Retired players reported moderate to very severe levels of depressive symptoms at nearly twice the rate of active players and at rates higher than those reported for the general population. Retired players suffered more from low social support from family and friends, and this was associated with greater depressive symptoms.
3.	Battochio & Stambulova [59]	Rookies *n* = 5 *M*_age_ = 22 Veterans n = 5 *M*_age_ = 29 Retirees *n* = 13 *M*_age_ = 47 All men	Canada and United States	Qualitative (interviews)	Coping strategies within different career stages	NHL players at an early career stage must deal with anxiety and different forms of pressure within the hockey context, such as roster spot uncertainty and performance pressure. Being able to cope through obstacles and setbacks leads to greater reward.
4.	Bruner et al. [60]	*n* = 8 (*M*_age_ = 17.2) All men	Canada	Qualitative (interviews)	Coping strategies within different career stages	On the ice, players thought it was challenging being a first-year player in the league because of low ice time and its impact on self-confidence. Off the ice, social support from teammates, billets, and family was found to be important to deal with stress reported by the participants.
5.	Dogusan & Koçak [61]	*n* = 21 Age range = 18 to 31 All women	Turkey	Qualitative (interviews)	Motivational construct	Participants reported that determination, ambition, being happy, self-confidence, and being passionate are all important factors provided by practicing ice hockey. Participants also reported that financial constraints, limited number of facilities, and fighting against gender stereotypes are obstacles to deal with.
6.	Gaudreau et al. [62]	*n* = 265 Age range = 13 to 20 *M*_age_ = 16.3 All men	Québec, Canada	Quantitative (survey)	Motivational constructs	Trajectories of positive affective states (high and decreasing; unstable; and medium and decreasing) and negative affective states (low and unstable; medium and unstable; and high and decreasing) were predicted by theoretically driven predictors assessed at the start of the season (self-determination, need satisfaction, athletic identity, and school identity).
7.	Géczi et al. [63]	*n* = 52 U18 *M*_age_ = 16.78 Adult *M*_age_ = 27.21 All men	Hungary	Quantitative (survey)	Anxiety and depression symptoms	The adult team scored lower on state anxiety, cognitive anxiety, and somatic A-state than the U18 team. Regarding the “peaking under pressure” factor, the adult team scored significantly higher than the U18 team, as well as in the “freedom of worry” factor.
8.	Géczi et al. [64]	*n* = 95 U16: n = 35 U18: n = 27 U20: n = 33 All men	Hungary	Quantitative (survey)	Anxiety and depression symptoms	Anxiety, stable personality traits, and coping skills for athletes were investigated. There were no differences among the age groups except for the trait anger scale results.
9.	Jaakkola et al. [65]	*n* = 265 *M*_age_ = 17.03 *SD* = 0.63 All men	Finland	Quantitative (survey)	Motivational constructs	A motivational climate that emphasizes effort, personal development, and improvement, as well as achievement goal mastering tasks, was a significant factor of enjoyment in junior ice hockey.
10.	Juntumaa et al. [66]	*n* = 1018 Age range = 14 to 16 All men	Finland	Quantitative (survey)	Motivational constructs	Mastery-oriented behavior positively influenced satisfaction in ice hockey. Authoritative parenting style was associated with adolescents’ high level of mastery-oriented behavior, but also the low level of task-irrelevant behavior and low level of norm-breaking behavior.
11.	Kiss & Nagy [67]	*n* = 293 Age range = 13 to 33 All men	Hungary	Quantitative (survey)	Motivational constructs	Four distinct motivational profiles were identified among elite young ice hockey players based on their self-determined motivation levels. Players with high self-determined motivation reported better coping skills and lower anxiety and perceived a more positive motivational climate.
12.	Lagimodiere & Strachan [68]	*n* = 5 Age range = 32 to 55 All men	Canada	Qualitative (interviews)	Coping strategies within different career stages	Retirement is seen as a difficult transition to cope with. Struggled to redefine personal identity following retirement. Identified a lack of social resources and support during retirement.
13.	Pankow et al. [69]	*n* = 6 (4 actives, 2 retirees) *M*_age_ = 32 *SD* = 5.32 All men	North America and Europe	Qualitative (interviews)	Coping strategies within different career stages	In minor hockey, participants developed interpersonal and individual psychological characteristics within a supportive social environment. During junior hockey, participants developed performance-oriented psychological characteristics and psychological skills arising from experiences of adversity and success. During professional hockey, they developed and refined performance-oriented psychological skills while also focusing on being a good teammate to promote their career longevity.
14.	Spink et al. [70]	*n* = 160 *M*_age_ = 18.6 *SD* = 1.1 All men	Canada	Quantitative (survey)	Motivational constructs	A positive psychological climate was linked to a higher perceived effort in athletes. If their roles were clear and they felt they could express themselves, athletes would exert more effort.
15.	Stambulova et al. [71]	*n* = 7 Age range = 18 to 30 All men	Sweden	Qualitative (interviews)	Coping within different career stages	From the data collection, authors generated a conceptual model of four phases that occurred during career transition: The preparation phase, orientation phase, adaptation phase, and stabilization phase. All stages were accompanied by demands, resources, barriers, coping strategies, and outcomes that athletes have to deal with, depending on which phase they are at.
16.	Stoeber et al. [72]	*n* = 138 14 or 15 years old All men	Finland	Quantitative (survey)	Motivational constructs	Measures of perfectionism and using the structural equation model (SEM) showed that perfectionistic strivings were associated with mastery-approach and performance-approach goals. Conversely, perfectionistic concerns were linked to mastery-avoidance, performance-approach, and performance-avoidance goals.
17.	Verner-Filion et al. [73]	*n* = 598 *M*_age_ = 16.56 *SD* = 1.41 All men	Québec, Canada	Quantitative (survey)	Motivational constructs	Athletes that were harmoniously passionate were associated with mastery- and performance-approach goals, which were also linked to higher life satisfaction, deliberate practice, and performance. On the other hand, obsessively passionate athletes were associated with performance-approach and performance-avoidance goals.

## Supplementary Materials

The following supporting information can be downloaded at: https://www.mdpi.com/article/10.3390/sports13070225/s1, File S1: PRISMA Extension for Scoping Reviews (PRISMAScR): Checklist and Explanation.

## Abbreviations

The following abbreviations are used in this manuscript:

APA	American Psychological Association
NHL	National Hockey League
PWHL	Professional Women’s Hockey League
SWLS	Satisfaction with Life Scale
