# Supplementary material for: Elite Ice Hockey Players’ Well-Being: A Scoping Review
Source: Sports (Basel). 2025 Jul 9;13(7):225. doi: 10.3390/sports13070225 (PMC12300323; doi:10.3390/sports13070225)
Supplement: Supplementary file 1 [file sports-13-00225-s001.zip › sports-3720561-supplementary.pdf]

Preferred Reporting Items for Systematic reviews and Meta-Analyses extension for Scoping Reviews (PRISMA-ScR) Checklist

SECTION	ITEM	PRISMA-ScR CHECKLIST ITEM	REPORTED ON PAGE #
**TITLE**			
Title	1	Identify the report as a scoping review.	P1 (See Title)
**ABSTRACT**			
Structured summary	2	Provide a structured summary that includes (as applicable): background, objectives, eligibility criteria, sources of evidence, charting methods, results, and conclusions that relate to the review questions and objectives.	P1 (See abstract)
**INTRODUCTION**			
Rationale	3	Describe the rationale for the review in the context of what is already known. Explain why the review questions/objectives lend themselves to a scoping review approach.	P4 : However, none of these studies focus specifically on elite ice hockey players. Moreover, ice hockey represents a particularly demanding sport due to its unique combination of physical, mental, and environmental challenges associated with its competitive context [17, 2]. The present review aims to fill the gap on athletes' well-being in this specific sport [55] and to explore the situation regarding this group of athletes in line with the transactional model of stress and coping developed by Lazarus and Folkman [32].
Objectives	4	Provide an explicit statement of the questions and objectives being addressed with reference to their key elements (e.g., population or participants, concepts, and context) or other relevant key elements used to conceptualize the review questions and/or objectives.	P4 : However, none of these studies focus specifically on elite ice hockey players. Moreover, ice hockey represents a particularly demanding sport due to its unique combination of physical, mental, and environmental challenges associated with its competitive context [17, 2]. The present review aims to fill the gap on athletes' well-being in this specific sport [55] and to explore the situation regarding this group of athletes in line with the transactional model of stress and coping developed by Lazarus and Folkman [32].
**METHODS**			
Protocol and registration	5	Indicate whether a review protocol exists; state if and where it can be accessed (e.g., a Web address); and if available, provide registration information, including the registration number.	P4 : This scoping review was developed based on the PRISMA ScR guidelines and consistent with the review protocol and the Arksey &

St. Michael's
Inspired Care.
Inspiring Science.

SECTION	ITEM	PRISMA-ScR CHECKLIST ITEM	REPORTED ON PAGE #
			O'Malley [56] five-step process, which involves: (1) identifying the research question; (2) identifying relevant studies; (3) study selection; (4) charting the data; and (5) collating, summarizing, and reporting the results.
Eligibility criteria	6	Specify characteristics of the sources of evidence used as eligibility criteria (e.g., years considered, language, and publication status), and provide a rationale.	P5: The inclusion criteria considered for this review included peer-reviewed articles published between 2002 and 2024. In addition, the interest in mental health in elite athletes and research involving hockey players has increased in the last 20 years. In these studies, potential participants had to be considered « elite », which means they were required to compete in professional leagues or in the most competitive leagues for adolescents ». Thus, participants had to be at least 14 years old. For example, the inclusion criterion was met by the M18 AAA[1], which features the best players (mostly between the age of 15 and 18 years old) in all Quebec's (Canada) hockey leagues. However, the inclusion criterion was not met for a high-school team, since the league level is not considered high enough. Articles on psychological factors and ice hockey players were selected. A few examples of psychological factors are the nature of significant childhood and adult relationships, the experience of ease or stress in social environments (e.g., school, work), and the experience of trauma [57].

[1] In Quebec (Canada) ice hockey, M18 AAA refers to the highest level of competitive play for male players under 18 years old, typically featuring elite athletes aiming for junior or professional careers.

SECTION	ITEM	PRISMA-ScR CHECKLIST ITEM	REPORTED ON PAGE #
			Finally, articles examining different sports using the same samples were not included unless conclusions could be drawn based solely on the sample of elite ice hockey players.
Information sources*	7	Describe all information sources in the search (e.g., databases with dates of coverage and contact with authors to identify additional sources), as well as the date the most recent search was executed.	P4: The initial search for potential articles was conducted May 11-30, 2022, generating a total of 476 articles. Because the writing took over a year, a second search was done on February 7, 2024, to include any possible articles added to the literature in 2023, as well as the beginning of the year of 2024. As shown in Figure 1, the following databases were systematically searched: Psycinfo (137), Sportdiscus (217) and Medline (321). Potential sources considered relevant in Google Scholar (9) were retrieved manually and also analyzed, bringing the total number of articles to 675. With the assistance of the university's specialized librarian, key words used for the literature search included the following: psychological factor* OR anxiet* OR motivation* OR *confidence OR "self concept" OR "self esteem" OR passion* OR pressure OR resilience OR "emotional intelligence" OR coping OR "psychological adjustment*" OR "well being" OR "well-being" OR wellbeing. Descriptors were also added to target specific concepts in the literature search: DE "performance anxiety" OR DE "Self-esteem" OR DE "self-confidence" OR DE

SECTION	ITEM	PRISMA-ScR CHECKLIST ITEM	REPORTED ON PAGE #
			“psychological resilience” OR DE “psychological stress.” Moreover, we used the key words “ice hockey” OR “hockey player*” to obtain and limit articles to ice hockey only. Because no advanced settings were available for Google Scholar, a manual search was conducted using the key words “psychological factors” and “ice hockey” within the 2002-2024 timeframe and was completed by consulting reference lists.
Search	8	Present the full electronic search strategy for at least 1 database, including any limits used, such that it could be repeated.	P4: The initial search for potential articles was conducted May 11-30, 2022, generating a total of 476 articles. Because the writing took over a year, a second search was done on February 7, 2024, to include any possible articles added to the literature in 2023, as well as the beginning of the year of 2024. As shown in Figure 1, the following databases were systematically searched: Psycinfo (137), Sportdiscus (217) and Medline (321). Potential sources considered relevant in Google Scholar (9) were retrieved manually and also analyzed, bringing the total number of articles to 675. With the assistance of the university’s specialized librarian, key words used for the literature search included the following: psychological factor* OR anxiet* OR motivation* OR *confidence OR "self concept" OR "self esteem" OR passion* OR pressure OR resilience OR "emotional intelligence" OR coping OR "psychological

SECTION	ITEM	PRISMA-ScR CHECKLIST ITEM	REPORTED ON PAGE #
			adjustment*" OR "well being" OR "well-being" OR wellbeing. Descriptors were also added to target specific concepts in the literature search: DE “performance anxiety” OR DE “Self-esteem” OR DE “self-confidence” OR DE “psychological resilience” OR DE “psychological stress.” Moreover, we used the key words “ice hockey” OR “hockey player*” to obtain and limit articles to ice hockey only. Because no advanced settings were available for Google Scholar, a manual search was conducted using the key words “psychological factors” and “ice hockey” within the 2002-2024 timeframe and was completed by consulting reference lists.
Selection of sources of evidence†	9	State the process for selecting sources of evidence (i.e., screening and eligibility) included in the scoping review.	P5: Regarding the article selection process, two authors (PLV and JD) independently reviewed all articles identified through the literature search, assessing their relevance based on predefined inclusion and exclusion criteria. Following the PRSIMA guidelines, each author initially selected potential articles for inclusion in the scoping review. Following this, they compared their selections and discussed the relevance of each article that passed the initial screening. In cases of disagreement, the authors revisited the specific articles and engaged in discussion to reach a consensus. Although a third author (SG) was available to arbitrate unresolved conflicts, no such intervention was necessary, as all disagreements were resolved

SECTION	ITEM	PRISMA-ScR CHECKLIST ITEM	REPORTED ON PAGE #
			through discussion between the two primary reviewers.
Data charting process‡	10	Describe the methods of charting data from the included sources of evidence (e.g., calibrated forms or forms that have been tested by the team before their use, and whether data charting was done independently or in duplicate) and any processes for obtaining and confirming data from investigators.	P6: Homemade analysis grids were used to provide a systematic method for reading articles in depth. The main characteristics of the 48 articles (study objective, population, scales and variables, statistical analysis, conclusion and limitations) were observed to validate their relevance. A column with the heading "Decision" was added to make the final decision. Overall, 11 articles were selected from the databases and five studies from Google Scholar, which led to the selection of 16 articles for the current scoping review that met every inclusion criterion. Various themes emerged from the studies, becoming subjects of further analysis to determine similarities and differences, as presented in Table 1 (See supplementary file 1).
Data items	11	List and define all variables for which data were sought and any assumptions and simplifications made.	P5-P6: Homemade analysis grids were used to provide a systematic method for reading articles in depth. The main characteristics of the 48 articles (study objective, population, scales and variables, statistical analysis, conclusion and limitations) were observed to validate their relevance. A column with the heading "Decision" was added to make the final decision. Overall, 11 articles were selected from the databases and five studies from Google Scholar, which led to the selection of 16 articles for the current scoping review that met every inclusion criterion. Various themes emerged from the studies, becoming subjects of further

SECTION	ITEM	PRISMA-ScR CHECKLIST ITEM	REPORTED ON PAGE #
			analysis to determine similarities and differences, as presented in Table 1 (See supplementary file 1).
Critical appraisal of individual sources of evidence§	12	If done, provide a rationale for conducting a critical appraisal of included sources of evidence; describe the methods used and how this information was used in any data synthesis (if appropriate).	N/A.
Synthesis of results	13	Describe the methods of handling and summarizing the data that were charted.	P6 : Thus, the selected articles were presented in a table to indicate their similarities and differences. Themes were identified to meet the objective of the scoping review regarding the implications for research and the theoretical framework. Consistent with the guidelines [55, 58], there was no quality evaluation of the selected studies. Results were interpreted by the authors and are described later in the article.
**RESULTS**			
Selection of sources of evidence	14	Give numbers of sources of evidence screened, assessed for eligibility, and included in the review, with reasons for exclusions at each stage, ideally using a flow diagram.	P6 : See Figure 1.
Characteristics of sources of evidence	15	For each source of evidence, present characteristics for which data were charted and provide the citations.	See Supplementary file 1
Critical appraisal within sources of evidence	16	If done, present data on critical appraisal of included sources of evidence (see item 12).	N/A
Results of individual sources of evidence	17	For each included source of evidence, present the relevant data that were charted that relate to the review questions and objectives.	See results section
Synthesis of results	18	Summarize and/or present the charting results as they relate to the review questions and objectives.	See results section
**DISCUSSION**			
Summary of evidence	19	Summarize the main results (including an overview of concepts, themes, and types of evidence available), link to the review questions and objectives, and	See discussion section

SECTION	ITEM	PRISMA-ScR CHECKLIST ITEM	REPORTED ON PAGE #
		consider the relevance to key groups.	
Limitations	20	Discuss the limitations of the scoping review process.	See discussion section
Conclusions	21	Provide a general interpretation of the results with respect to the review questions and objectives, as well as potential implications and/or next steps.	See conclusion section
**FUNDING**			
Funding	22	Describe sources of funding for the included sources of evidence, as well as sources of funding for the scoping review. Describe the role of the funders of the scoping review.	N/A

JBI = Joanna Briggs Institute; PRISMA-ScR = Preferred Reporting Items for Systematic reviews and Meta-Analyses extension for Scoping Reviews.
* Where *sources of evidence* (see second footnote) are compiled from, such as bibliographic databases, social media platforms, and Web sites.
† A more inclusive/heterogeneous term used to account for the different types of evidence or data sources (e.g., quantitative and/or qualitative research, expert opinion, and policy documents) that may be eligible in a scoping review as opposed to only studies. This is not to be confused with *information sources* (see first footnote).
‡ The frameworks by Arksey and O'Malley (6) and Levac and colleagues (7) and the JBI guidance (4, 5) refer to the process of data extraction in a scoping review as data charting.
§ The process of systematically examining research evidence to assess its validity, results, and relevance before using it to inform a decision. This term is used for items 12 and 19 instead of "risk of bias" (which is more applicable to systematic reviews of interventions) to include and acknowledge the various sources of evidence that may be used in a scoping review (e.g., quantitative and/or qualitative research, expert opinion, and policy document).

From: Tricco AC, Lillie E, Zarin W, O'Brien KK, Colquhoun H, Levac D, et al. PRISMA Extension for Scoping Reviews (PRISMAScR): Checklist and Explanation. Ann Intern Med. 2018;169:467–473. doi: 10.7326/M18-0850.
